# Diagnostic Performance of a DOAC Urine Dipstick in Obese Outpatients with Atrial Fibrillation: Comparison with Plasma Concentrations

**DOI:** 10.3390/jcm15020466

**Published:** 2026-01-07

**Authors:** Arianna Pannunzio, Valentina Castellani, Erminia Baldacci, Vittoria Cammisotto, Rosaria Mormile, Ilaria Maria Palumbo, Nicola Porcu, Antonio Chistolini, Graziella Bernardini, Danilo Menichelli, Daniele Pastori, Job Harenberg, Francesco Violi, Pasquale Pignatelli

**Affiliations:** 1 Department of General Surgery, Surgical Specialty and Anaesthesiology, Sapienza University of Rome, 00185 Rome, Italy; arianna.pannunzio@uniroma1.it (A.P.);; 2 Department of Medical and Cardiovascular Sciences, Sapienza University of Rome, 00185 Rome, Italypasquale.pignatelli@uniroma1.it (P.P.); 3 Department of Translational and Precision Medicine, Sapienza University of Rome, 00185 Rome, Italy; 4 U.O.S.D. Medicina Rigenerativa DAI, Medicina Diagnostica e Radiologia, Policlinico Umberto 1, 00161 Roma, Italy; 5 Istituto di Ricovero e Cura a Carattere Scientifico (IRCCS) Neuromed, 86077 Pozzilli, Italy; 6 Medical Faculty Mannheim, Ruprecht Karls University of Heidelberg, 69117 Heidelberg, Germany

**Keywords:** obesity, non-valvular atrial fibrillation, DOACs, urine dipstick, plasma concentrations

## Abstract

**Background**: atrial fibrillation (AF) patients with obesity and high thromboembolic risk need oral anticoagulant therapy. Limited data are available on direct oral anticoagulants (DOACs) in this population, and a point-of-care method has been validated to support rapid clinical decisions and to identify on-off plasma concentration thresholds. **Methods**: This is a monocentric, cross-sectional diagnostic accuracy study on obese AF outpatients referred to Policlinico Umberto I of Rome. Urinary Dipsticks were assessed with separate pads for factor Xa (FXA-i) and thrombin inhibitor (THR-i) and compared to the reference standard of trough and peak plasma concentrations with chromogenic assays/dTT and prespecified plasma thresholds for each DOAC. Study endpoints were the sensitivity, specificity, positive and negative predictive values (PPV and NPV) of DOACs Dipstick compared to plasma concentrations. Sub-analyses according to obesity severity and type of DOAC were performed. **Results**: 320 paired plasma and urine samples were available from 160 enrolled patients (mean age 73.2 ± 9.1 years). Compared to trough plasma concentrations, DOACs Dipstick showed a sensitivity of 99.24% (mean, 95% confidence interval, CI 95.82–99.98), specificity of 6.89% (0.85–22.76), PPV 82.80% (81.32–84.18), NPV 66.67% (15.79–95.52). On the other hand, compared to peak plasma concentrations, DOACs Dipstick showed a sensitivity of 97.8% (93.7–99.5), specificity of 0% (0.0–15.4), and PPV of 85.9% (85.6–86.2). Urinary Dipstick showed a sensitivity of 99.10% (95.4–100.0), specificity of 4.70% (0.60–16.20) and a PPV and NPV of 74.50% (73.2–75.8) and 66.70 (15.7–95.6), compared to plasma thresholds > 30 ng/mL of FXA-I and THR-I. Sub-analyses showed similar results between FXA-i and THR-i. **Conclusions**: The urine point-of-care has high sensitivity, acceptable PPV, but low specificity and NPV in AF obese patients and may be useful only in selected clinical scenarios.

## 1. Introduction

Obesity is defined by the World Health Organization (WHO) as abnormal or excessive fat accumulation, which presents a risk to health [1]. Its prevalence has been increasing worldwide in recent decades, reaching pandemic proportions [2,3] and varying according to the specific country: from <6% in Japan [3], to >20% in the United States [4] and in Europe [1,5]. Obesity is a risk factor for diabetes [6], cancer [7], venous thromboembolism [7,8,9] and almost all cardiovascular diseases (CVDs), including atrial fibrillation (AF) [10], both for first events and recurrences. This association is important since AF is the most common arrhythmia worldwide [11], and because the prescription of anticoagulant therapy in obese patients with AF may be challenging [11]. Anticoagulant therapy in patients with non-valvular AF can be provided with direct oral anticoagulants (DOACs) or Vitamin K Antagonists (VKAs). Currently, appropriate VKA efficacy is assessed by periodic monitoring of international normalized ratio (INR), while DOAC plasma concentration is not routinely required since the DOAC dosing is standard. Concern has been raised about DOACs efficacy and safety, especially in patients with obesity, since data from pharmacokinetic (PK) and pharmacodynamic (PD) studies are scarce [11]. Recent guidelines suggest assessing plasma DOAC concentrations in obese patients > 120 kg or with a BMI > 40 kg/m^2^ due to potential abnormalities in PK and volume distribution [11,12,13,14]. Chromogenic substrate assays are available to measure DOACs plasma concentrations but are not routinely used in clinical practice [15].

Recently, a DOAC point-of-care rapid urine Dipstick was introduced in clinical practice. The advantages of using the Dipstick to evaluate DOACs in patients with AF and obesity include having the result available within 15 min, being less expensive and widely available, especially during an outpatient follow-up visit. Current studies suggest a potential use of DOAC point-of-care rapid urine Dipstick during emergency medical situations with severe bleeding or thrombotic events and before urgent major surgical interventions to accelerate medical decision-making. Paired urine and plasma samples of patients with acute stroke in the emergency setting showed a high sensitivity and specificity of factor Xa and thrombin inhibitors by the specific pads of DOAC Dipstick compared to liquid chromatography mass spectrometry and chromogenic substrate assay results at a clinically relevant >30 ng/mL plasma threshold and results were available faster compared to a chromogenic substrate assay [16,17]. However, the DOAC point-of-care rapid urine dipstick has not been investigated in a specific population, such as obese patients with AF, in whom a PK alteration may be observed, and its role in specific settings, such as outpatient clinics, remains unclear.

For this reason, the aim of the present study was to perform a diagnostic accuracy study aiming to assess the sensitivity, specificity, positive and negative predictive values (PPV and NPV) of the DOAC point-of-care rapid urine Dipstick compared to trough and peak plasma concentrations of DOACs in AF outpatients with obesity. The primary outcome was to compare the DOAC point-of-care rapid urine Dipstick with a pre-specified specific threshold for each of the DOAC according to international guidelines [18,19]; the secondary outcome was to compare the point-of-care rapid urine Dipstick test with the 30 ng/mL threshold [19].

## 2. Methods

**Figure 1:**
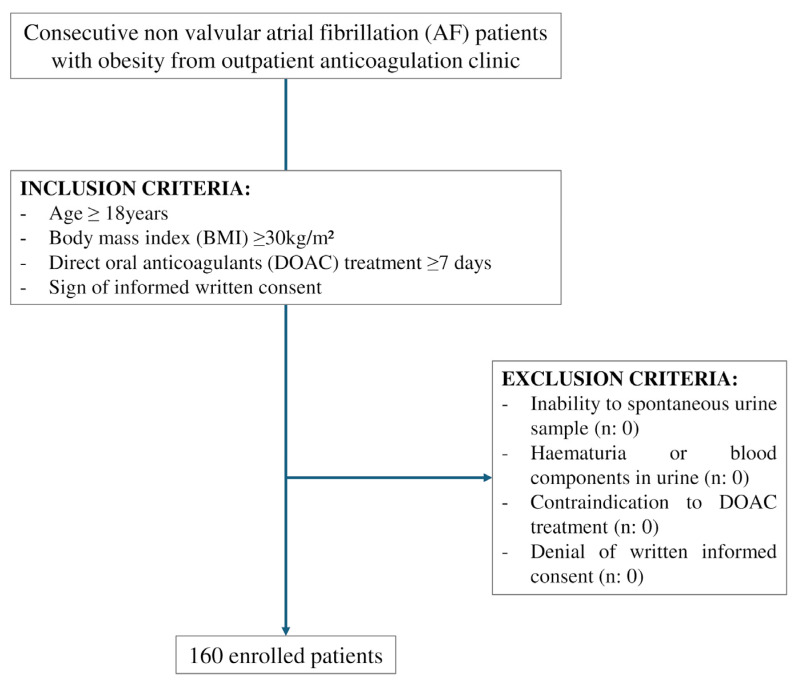
Flow diagram.

In this observational, cross-sectional, monocentric, diagnostic accuracy study, obese outpatients with non-valvular AF in DOACs treatment were enrolled. The study was promoted by the Department of Medical and Cardiovascular Sciences of Sapienza University of Rome, and patients referred to the outpatient anticoagulation clinic of Policlinico Umberto I were enrolled consecutively from 2022 to 2024. Inclusion criteria were patients aged ≥18 years, with a Body Mass Index (BMI) ≥ 30 kg/m^2^, receiving a DOAC for non-valvular AF for at least 7 days, and who provided informed written consent. Exclusion criteria were denial of written informed consent, inability to provide a spontaneous urine sample, any contraindication to the administration of DOACs and conditions with known haematuria and blood components such as urobilinogen (see Figure 1). The last exclusion criterion was that the alteration of urine colour may compromise the interpretation of the pad colours of the DOAC Dipstick.

All patients signed informed written consent prior to study entry. The study was approved by the local ethics committee of Sapienza University (No. 0234/2022—approval date: 23 March 2022) and was conducted according to the 1975 Declaration of Helsinki.

### 2.1. Calculation of Sample Size

According to a previous study performed on 260 measurements [20], 160 patients needed to be recruited.

### 2.2. Definition of Obesity

The WHO classifies adult obesity using the BMI with specific cut-offs. The BMI is measured by calculating [(weight in kilograms)/(height in metres)^2^] and is a simple index intended to classify adults within one of three categories [21]: underweight if the BMI is ≤18.5 kg/m^2^, overweight if the BMI between 25 and 29.9 kg/m^2^ or obese if the BMI ≥ 30 kg/m^2^. In the obesity category, there is a subclassification based on the degree: class I obesity has a BMI between 30 and 34.9 kg/m^2^; class II obesity has a BMI between 35 and 39.9 kg/m^2,^ and class III obesity has a BMI ≥ 40 kg/m^2^ (see Supplementary Table S1 in Supplementary Materials).

### 2.3. Blood Sampling of DOAC and Plasma Concentrations Assessment

Blood samples from AF patients were collected in tubes with anticoagulant (3.8% sodium citrate) immediately before the administration of the last dose of DOAC to assess the trough plasma concentration and 2 h after DOAC intake to assess the peak plasma concentration. The tubes were centrifuged at 1500× *g* for 10 min at room temperature to obtain platelet-poor plasma samples, which were then immediately stored at −80 °C until analyzed. For a more detailed description of DOACs plasma concentrations assessment, which has also been described previously [21], see “Supplementary Methods” in Supplementary Materials.

As reference values, data from EMA technical reports can be considered [22]. No EMA reference data are available for edoxaban and those reported in the literature are limited [23]. Ruff et al. reported in a large study the expected trough concentrations after administration of 30 or 60 mg per day of edoxaban [12]. The reference value of trough and peak plasma concentrations of our cohort has been previously reported [21,22] and is summarized in Supplementary Table S2.

### 2.4. DOAC Dipstick

For laboratory evaluation of DOAC, urine specimens were collected in 10 mL BD Vacutainer tubes (BD Diagnostics, Plymouth, UK) immediately (10 to 15 min) after collection of blood and were analyzed within 2 h. The test strip contains separate pads containing immobilized reagents specific for FXA and thrombin inhibitors. The test strips also contain a pad without reagents for the determination of urine colour because abnormal urine colour may interact with the colours of the FXA and thrombin pads. A fourth pad determines creatinine in urine because a reduced elimination of creatinine may occur in patients with renal impairment, leading also to a reduced elimination of DOACs. The test strips were incubated for 2 to 3 s into the urine samples, ensuring that all pads were completely embedded in urine. The immobilized reagents on the test pads react for 10 min with DOACs present or absent in urine.

The test strip contains two pads containing immobilized coagulation enzymes Xa or thrombin and specific factor Xa or thrombin-specific substrates. Upon a 2 to 3 s incubation time of the test strips with urine, the enzymes released the chromophore from the substrate in an amount that was dose dependent on the amount of DOAC present in the urine. The colours of the chromogens were different for oral direct factor Xa and thrombin inhibitors. Following a 10 min incubation time of urine on the test pads, the colour on the test pads was read by DOASENSE^®^ Reader (Heidelberg, Germany), an instrument designed to measure the above-mentioned parameters. The instrument uses LEDs with specific wavelengths as light sources to illuminate the corresponding measuring surfaces of the pads. A photodiode detected the reflected light. For analysis by the reader, the DOAC Dipstick test strip was dipped into the urine sample and placed on the test strip holder of the instrument. The integrated detector recognized the pads of the test strip, and after 10 min, the intensity of the reflected light was measured. The microcontroller converted the intensity of the reflected light into an analytical value. The corresponding qualitative result was displayed on the screen and printed by the integrated thermal printer. The result was displayed as negative (‘NEG’) or positive (‘POS’) for factor Xa and thrombin inhibitors and as normal (‘norm’) or low (‘low’) for creatinine [24]. The DOASENSE^®^ Reader was used exclusively (without the combination of visual reading), and there was no interobserver variability assessment. Assessors of urine DOACs Dipstick were blinded to chromogenic anti-Faa assays and dTT results.

The test has already demonstrated a low interindividual variability in visual interpretation of the Xa inhibitors (Xa-i) and IIa inhibitor (IIa-i) pads in relation to the presence of direct oral factor Xa inhibitors (DXIs) and direct thrombin inhibitors (DTIs) [24]. Conditions like haematuria and blood components such as urobilinogen alter the colour of urine and may compromise the interpretation of pad colours. To address this, the DOAC Dipstick contains a pad that detects abnormal urine colour. The normal yellow colour of urine does not influence the white colour of this pad, because a low volume of urine is absorbed. The specific colours of the test strip also detect creatinine in the urine, which shows whether DOAC excretion is reduced, suggesting renal impairment [25,26].

### 2.5. Study Endpoints

The primary endpoint of the study was to establish the sensitivity, specificity, PPV and NPV and accuracy of the Dipstick urinary test (index test) compared to the trough plasma concentrations threshold (reference test) according to international guidelines and recommendations [18] in the overall population and according to factor Xa and factor IIa inhibitors.

As secondary endpoints, we evaluated the sensitivity, specificity, PPV and NPV of the Dipstick urinary test (index test) compared to international guidelines peak plasma concentrations threshold (reference test) [18,19] and compared to 30 ng/mL threshold through plasma concentration [19]. This cut-off was taken from a previous study, which demonstrated that comparing DOAC Dipstick pads with liquid chromatography-tandem mass spectrometry for factor Xa and factor IIa inhibitors in urine samples, the sensitivity, specificity, accuracy, NPV and PPV values and agreement between methods for determination of factor Xa inhibitors were at least non-inferior to 95% with a 0.5% margin and of factor IIa inhibitors superior to 97.5% [27].

Primary and secondary outcomes were conducted per-patient. Prespecified subgroup analyses were conducted according to the type of DOAC, obesity class and body weight (≥120 kg or <120 kg).

### 2.6. Statistical Analysis

Categorical variables were reported as percentages, continuous variables were reported as mean ± standard deviation (SD) or median and interquartile ranges (IQR) based on the distribution, which was tested with the Kolmogorov–Smirnov test. Differences between percentages were evaluated with the chi-square test and Fisher’s exact test. Student’s *t*-test and Pearson correlation analysis were used for normally distributed continuous variables. Appropriate non-parametric tests were used for all the other variables.

An initial descriptive analysis was performed to evaluate the clinical and biochemical characteristics of the population according to the class of DOAC, DXIs or DTIs. Subsequently, the sensitivity, specificity, PPV, NPV, and accuracy values with their binomial 95% confidence interval (95% CI) of the urinary test strip were calculated for the qualitative DOAC Dipstick’s Factor Xa and thrombin pad results versus the trough and peak plasma threshold concentration. These parameters were considered valid if not lower than 95% with a margin of 0.5% compared to the plasma test regarding factor Xa inhibitors and if higher than 97.5% for the inhibitor of thrombin. Low doses of DOACs were defined and prescribed according to European Society of Cardiology guidelines [18].

In the sub-analysis, we analyzed the sensitivity, specificity, PPV and NPV values and accuracy of urinary Dipstick separately for peak and trough values, and according to grade I and ≥II obesity.

Furthermore, analyses were also performed according to the type of DOAC, evaluating factor Xa inhibitors (apixaban, edoxaban, and rivaroxaban) and, separately, direct thrombin inhibitors (dabigatran) and according to body weight ≥ 120 kg or <120 kg.

Statistical significance was established at a value of *p* < 0.05. The analyses were performed with the IBM SPSS Software Version 25 for Windows^®^ and with MedCalc (Version 18.2.1) for Windows^®^.

## 3. Results

### 3.1. Study Population

The study enrolled 160 patients with AF and obesity, with 54 (33.8%) women and a mean age of 73.2 ± 9.1 years, including samples obtained before and 2 h after intake of the morning dose of the DOAC. Overall, we found 130 true positives, 27 false positives, 1 false negative, 2 true negatives. We found a low number of negative tests (n: 3): this could limit the interpretation of NPV, likelihood ratios and specificity, which may result in more imprecise interpretations. The main characteristics of the population according to the type of DOAC (anti-Xa or anti-IIa inhibitors) are represented in Table 1. 26 (16.3%) patients were treated with dabigatran, 134 (83.8%) with inhibitors of factor Xa (n: 35 rivaroxaban, n: 75 apixaban, n: 24 edoxaban). There were no statistically significant differences between the two groups (factors Xa and thrombin inhibitors) with respect to the cardiovascular risk factors and to the concomitant drugs assumed, apart from previous stroke or TIA, which was more frequent in patients receiving anti-Xa inhibitors (*p* = 0.002) (Table 1). There was no difference in the positivity of the urine dipstick test between the two groups (92.3% vs. 95.5%; *p* = 0.491). Regarding the BMI, more patients with class I of obesity were assumed to be on anti-Xa inhibitors, while anti-IIa inhibitors were more frequent in class II obesity (*p* = 0.026) (Table 1).

### 3.2. Primary Outcome

#### 3.2.1. Point-of-Care Sensitivity, Specificity, Accuracy Compared to Trough Plasma Concentrations

Overall, among 160 samples collected during trough plasma concentrations, there were 130 true positives, 27 false positives, 1 false negative and 2 true negatives. The Dipstick showed a sensitivity of 99.24% (95% CI: 95.82–99.98), specificity of 6.89% (95% CI: 0.85–22.76), PPV 82.80% (95% CI: 81.32–84.18) and NPV 66.67% (95% CI: 15.79–95.52) as reported in Table 2. Specificity was extremely low in primary and secondary endpoints, as well as in subgroup analyses, mainly due to the very small number of true negative samples.

No significant differences are found between true positives and false positives on comorbidities, therapy and clinical features (Table 3).

#### 3.2.2. Point-of-Care Sensitivity, Specificity, Accuracy Compared to Trough Plasma Concentrations According to Anti-Xa Inhibitors and Anti-IIa Inhibitors

In patients treated with anti-Xa inhibitors, DOAC urinary Dipsticks showed high sensitivity (100.0%, 95% CI 79.4–100.0) but low specificity (10%, 95% CI 0.25–44.5). On the other hand, anti-IIa inhibitors showed similar sensitivity (99.1%, 95% CI 95.3–99.9) but lower specificity (5.3, 95% CI 0.13–26.0). Sensitivity, specificity, PPV, NPV and negative and positive likelihood ratios of DOACs urinary Dipstick according to anti-Xa and anti-IIa inhibitors are reported in Table 4.

### 3.3. Secondary Outcome

#### 3.3.1. Point-of-Care Sensitivity, Specificity, Accuracy Compared to Peak Plasma Concentration

Evaluating the accuracy of urine Dipstick compared to peak plasma concentrations, the Dipstick showed a high sensitivity (97.8%, 95% CI 93.7–99.5) and a low specificity (0%, 95% CI 0.0–15.4). Further details are reported in Supplementary Table S3.

#### 3.3.2. Point-of-Care Sensitivity, Specificity, Accuracy Compared to Plasma Concentration Threshold > 30 ng/mL

Urinary Dipstick, compared to plasma concentrations threshold > 30 ng/mL, showed a high sensitivity (99.1%, 95% CI 95.4–100.0) but a low specificity (4.7%, 95% CI 0.6–16.2). PPV, NPV, negative and positive likelihood ratios, and accuracy are reported in Table 5.

### 3.4. Subgroup Analyses

#### 3.4.1. Subgroup Analysis According to Type of DOAC

Performing a subgroup analysis according to the type of DOAC, all DOACs showed a high sensitivity between 99.1% and 100% and a low specificity (0–33.3%). Rivaroxaban seems to be the DOAC with the highest specificity (33.3%) and sensitivity (100%), while edoxaban showed only a high sensitivity (100%). Sensitivity, specificity, accuracy and positive and negative likelihood ratios of this sub-analysis are shown in Supplementary Table S4.

Results regarding the number of true positive, false positive, true negative and false negative according to trough and peak plasma concentration and according to each DOAC are shown in Supplementary Table S5.

Evaluating inhibitors of FXa and thrombin DOAC Dipstick separately and comparing DOAC Dipstick to the trough plasma concentration threshold ≥30 ng/mL, we found a higher NPV and specificity in patients taking FXa inhibitors compared to thrombin inhibitors (Supplementary Table S6, Panel A). Conversely, no significant difference was observed between DOAC Dipstick and peak plasma concentrations in both groups of FXa and thrombin inhibitors (Supplementary Table S6, Panel B).

#### 3.4.2. Subgroup Analysis According to Obesity Class and Body Weight

We performed a subgroup analysis according to obesity class I or ≥2 as shown in Supplementary Table S7. In all degrees of obesity, the urinary Dipstick showed a high sensitivity and a low specificity, confirming the results of the main analysis. Results about the number of true positive, false positive, true negative and false negative according to obesity class and type of DOAC (Factor Xa or dabigatran) are reported in Supplementary Table S8.

Finally, we performed a subgroup analysis according to weight < 120 kg or ≥120 kg as shown in Supplementary Table S9. In both categories, the urinary Dipstick showed a high sensitivity and very low specificity.

All prespecified subgroup analyses confirmed the results of the primary outcome, namely that the point-of-care urinary Dipstick exhibits high sensitivity and positive predictive value, and low specificity and negative predictive value.

## 4. Discussion

In this study, we examined the sensitivity, the specificity, the PPV and the NPV of the DOAC Dipstick test in outpatients with AF and obesity. The Dipstick urine test had a high sensitivity and positive predictive value, but low specificity and negative predictive value. In this way, if the test is positive, there is an 82.80% probability that the trough DOAC plasmatic concentration is in range; if the test is negative, we do not have enough data to establish if the trough DOAC plasmatic concentration is below range, due to the low number of total negative tests.

Obese patients undergoing anticoagulant treatment have several concerns; the treatment with VKAs for the reduction in thromboembolic risk of AF may require high doses of anticoagulant and extended treatment initiation periods to achieve therapeutic INR levels [28]. On the other hand, DOACs had a standard administration dose, but it is unclear if they had an on-range plasma concentration in patients with higher distribution volume, such as obese patients. For this reason, recent guidelines suggest using plasmatic DOAC concentration in obese patients with a weight of ≥120 kg or with a BMI ≥ 40 kg/m^2^ [11,12,13,14]. Furthermore, recent studies showed a higher risk of thrombotic and bleeding events according to baseline DOAC plasma concentrations [29,30]. However, a method to measure DOACs plasma concentrations has been validated but is not of routine use in clinical practice (14) due to poor distribution, need for time to process the result and challenges in standardization for each DOAC. For this reason, the choice of a rapid and widely available test, such as a urine Dipstick, may be helpful in these patients.

Previous studies about the DOAC Dipstick are heterogeneous in terms of therapeutic indication for the anticoagulant therapy, DOACs analyzed and characteristics of the enrolled population. In particular, the clinical setting (outpatients or emergency care), indications for oral anticoagulant therapy [AF and venous thromboembolism (VTE)] and plasmatic thresholds of DOACs considered for Dipstick positivity are mixed.

In a prospective observational cohort study [31] performed on outpatients taking DOACs, the presence of DXIs in urine samples was independently evaluated by visual interpretation of the DOAC Dipstick pad colours. Positive DOACs Dipstick results were compared with a threshold plasma DOAC concentration of ≥30 ng/mL. Among 120 patients, the prescribed DOACs were rivaroxaban and apixaban, and the reasons for anticoagulant therapy were both AF and VTE. The DOAC Dipstick test had high sensitivity (97.2%) and positive predictive value (89.5%). However, results could not be extended to all DOACs, and specificity and negative predictive value could not be determined due to the low number of true negative values and for this reason, they were not reported.

Another prospective single-centre study enrolled 128 patients [25] in different clinical settings, highlighting that the negative predictive values and sensitivities for anti-Xa inhibitors and anti-IIa inhibitors Dipstick were 100% at 30 ng/mL plasma, for specificities of 6 and 21% and for positive predictive values of 62 and 72%, respectively. Nevertheless, no patient was assuming edoxaban, so not all the anti-Xa inhibitors were evaluated. The clinical indication for assuming DOAC were for both AF and VTE, and a creatinine clearance < 30 mL/min was an exclusion criterion. The plasma concentration cut-off was established at 30 ng/mL because in a perioperative setting it was suggested that this drug level may justify postponing a high bleeding risk operation or to administer a reversal agent before surgical intervention, but in case of unavailability of the test and a short turn-around time a negative DOAC Dipstick test result would justify with holding a reversal agent for DXI and DTI and to perform urgent major surgery in patients [16].

Regarding plasmatic thresholds, there is still some debate about which plasma DOAC concentrations are clinically relevant; both 30 and 50 ng/mL have been reported as clinically relevant to support decision-making processes [17]. Nevertheless, most of the studies considered 30 ng/mL, and for this reason, we performed the subgroup analysis choosing this cut-off. Ord et al. also chose 30 ng/mL as a threshold in a study which aimed to compare plasma concentration and urine Dipstick in patients taking DOACs and controls not taking DOACs [32]. DOAC Dipstick test results were positive in 21/23 patient urine samples at a plasma DOAC concentration of ≥30 ng/mL and in 2/23 patient urine samples at a plasma DOAC concentration of <30 ng/mL. Inter-observer agreement was above 90% for visual analysis of urine samples and was 100% for the Dipstick reader analysis of patient and control group urines.

Of great relevance, in none of the above-mentioned studies was BMI specified among the clinical characteristics of the population.

In the only meta-analysis available on Dipstick test, Harenberg et al. analyzed five clinical studies, demonstrating a high sensitivity (97.8% with 95% CI 95.6–99.0 for DXIs and, 98.3% with 95% CI 91.0–100 for DTIs), an acceptably high negative predictive value (86.6% with 95% CI 76.0–93.7 for DXIs and 99.6% with 95% CI 97.7–100), but low specificity (50.0% with 95% CI 40.2–59.0 for DXIs) when compared with levels measured with liquid chromatography tandem mass spectrometry or calibrated chromogenic assays to reliably exclude plasma DOAC concentrations > 30 ng/mL [19].

### 4.1. Clinical Implications

Our study has clinical implications. Firstly, we found high positive tests with a high proportion of false positives. In the obese population, who could be exposed to a lower serum concentration of DOAC due to high volume of distribution, a false positive test may prevent the physician from identifying patients with DOAC concentrations that require a change in anticoagulant treatment. In this way, obese patients could be exposed to an increased risk of thromboembolic stroke due to inaccurate anticoagulant therapy. For this reason, it could be reasonable to confirm the results with a plasma assay if a clinical decision is required on anticoagulant treatment.

In addition, our findings suggest a potential role of the Dipstick as a rapid screening tool to confirm exposure in contexts where any detectable DOAC is fundamental (e.g., surgical or emergency settings). However, its use is limited by negative results, given the low NPV and scarcity of negatives.

Overall, the urine DOAC Dipstick seems to be more helpful for confirming clinically relevant DOAC exposure than for safely excluding subtherapeutic levels and may be useful in a particular clinical setting, such as preoperative assessment and emergency settings, rather than routine use in obese outpatients with AF.

Finally, our study does not provide evidence regarding clinical outcomes such as bleeding or thromboembolic events. Owing to the study design, we did not investigate the predictive role of the urine Dipstick for clinical outcomes. Further prospective studies are needed to clarify whether urine dipstick testing may have prognostic value in this patient population.

### 4.2. Strengths and Limitations

Our study is the first to explore the Dipstick performance in a selected population of patients with AF and different degrees of obesity, treated with all available DOACs. This clinical setting is characterized by limited real-world data; consequently, current guidelines and consensus rely on low-grade evidence. Moreover, it is the only study which used pre-specified plasmatic cut-offs according to the specific DOAC, dosing regimen and indication and evaluated their correspondence with Dipstick positivity, as advised by EMA technical reports [22].

However, several limitations should be acknowledged.

Firstly, it is a monocentric cross-sectional study which included only Caucasian patients with a relatively small proportion in class III of obesity. In addition, due to sample size, our study could not adequately explore the role of Dipstick in clinical subgroups of patients, such as patients with severe chronic kidney disease or with concomitant interfering drugs.

In addition, the number of false positives and the specificity of the test are major limitations to the applicability of the test in obese outpatients with AF. In particular, the low NPV and specificity, due to a high number of false positives and the high sensitivity of the test, represent a major limitation of urine Dipstick in the outpatient clinical setting to evaluate obese patients with a potential low serum DOAC concentration.

Furthermore, the limited number of negative tests constrains the interpretation of the test’s low specificity and negative predictive value. Nevertheless, this limitation stems from the high proportion of false-positive results. This issue warrants further investigation in future studies. In addition, the low number of negative tests could limit the interpretation of NPV and specificity, which could be imprecise, restricting the external validity and reproducibility.

Additionally, the present study did not assess clinical outcomes, in terms of bleeding or thromboembolic events, linked to Dipstick-guided decisions. This is a cross-sectional diagnostic study without any follow-up.

Several limitations also exist in DOAC Dipstick testing. There is less apixaban excreted in urine than rivaroxaban or edoxaban, consistent with apixaban’s primary hepatic clearance mechanism. Detailed data on the appearance of DOACs in plasma and urine following oral intake, and the influence of active DOAC metabolites on the urine Dipstick test, remain limited [33]. In addition, it should be investigated whether the method of urine collection may affect the performance of the Dipstick, considering the time of the last bladder emptying. This information is likely to be unknown in most cases of dipstick use, reflecting the pragmatism of the study design, but may be addressed as part of a follow-up study.

Finally, the distribution of DOACs in our cohort was centre-specific and may not be representative of other institutions and settings [19]. Given that our population consisted exclusively of obese outpatients with AF, the results cannot be generalized to other patient populations or clinical contexts.

### 4.3. Future Prospectives

Although urinary DOAC Dipsticks seem to have low NPV and specificity in obese AF patients, it is still unclear if clinical variables such as timing of last dose and renal function could improve decision-making, and no studies on this topic have been performed. Probably the high volume of distribution and the hyperfiltration that are usually observed in obese patients contribute to our results, but no robust evidence is still available. Therefore, further studies are warranted to assess the role of urine DOAC Dipstick in selected populations and to evaluate how clinical and pharmacokinetic variables may influence its diagnostic performance.

## 5. Conclusions

In conclusion, this study demonstrates that the Dipstick urine test has high sensitivity, an acceptable PPV, but extremely low specificity and NPV in obese outpatients with AF, compared with plasma DOAC concentrations, including at the 30 ng/mL threshold. These findings suggest that the test may be useful in well-defined clinical settings, in which high-test sensitivity and rapid assessment can help the physician make rapid decisions (e.g., Emergency Room). However, it does not appear suitable for routine outpatient clinical settings, especially in special populations such as obese patients requiring anticoagulation. Studies with a larger sample of patients are needed to examine factors affecting the performance of the test and to clarify its role in clinical decision-making.

## Figures and Tables

**Table 1:** Characteristics of patients according to anti-Xa inhibitors (anti-Xa I) and anti-thrombin inhibitors (anti-IIa I) anticoagulants.

	Anti-IIa I (n: 26)	Anti-Xa I (n: 134)	Overall (n: 160)	*p*-Value
***Age (mean)***	73.4 ± 7	73.1 ± 9	73.1 ± 9	0.718
***Female (%)***	23.1	35.8	33.8	0.209
***Dipstick positive (%)***	92.3	95.5	95.0	0.491
***Hypertension (%)***	96.2	94.8	95.0	0.768
***Diabetes (%)***	46.2	38.1	39.4	0.439
***Previous MI (%)***	34.6	18.7	21.3	0.069
***Previous stroke/TIA (%)***	38.5	13.4	17.5	0.002
***Smoke (%)***	7.7	12.7	11.9	0.471
***Ex-smoke (%)***	57.7	41.0	43.8	0.117
***COPD (%)***	19.2	20.9	20.6	0.848
***PAD (%)***	15.4	5.2	6.9	0.061
***HF (%)***	34.6	21.6	23.8	0.155
***Liver disease (%)***	11.5	11.9	11.9	0.954
***Cancer (%)***	11.5	17.2	16.3	0.477
***BMI < 35 (%)***	57.7	76.9	73.8	0.042
***BMI ≥ 35 (%)***	42.3	23.1	26.3	
***CHA_2_DS_2_-VASc Score***	4.5 ± 1.8	3.8 ± 1.5	3.9 ± 1.6	0.099
***HAS-BLED score***	1.3 ± 0.8	1.2 ± 0.7	1.2 ± 0.7	0.344
***Therapy***				
***Antiplatelet (%)***	0.0	4.5	3.8	0.271
***ACE inhibitors/ARBs (%)***	84.6	70.9	73.1	0.149
***Nitro-derivates*** ***(%)***	0.0	3.0	2.5	0.372
***Beta-blockers (%)***	73.1	74.6	74.4	0.868
***Digoxin (%)***	11.5	9.0	9.4	0.679
***Antiarrhythmics (%)***	11.5	23.9	21.9	0.164
***Amiodarone (%)***	3.8	6.0	5.6	0.667
***Statins (%)***	76.9	74.6	75.0	0.805
***Ezetimibe (%)***	26.9	23.9	24.4	0.741
***SSRI (%)***	11.5	6.7	7.5	0.393
***Diuretics (%)***	61.5	64.2	63.7	0.798
***PPI (%)***	53.8	61.9	60.6	0.439
***Allopurinol (%)***	7.7	19.4	17.5	0.150
***Clinical Features***				
***BMI (continuous)***	33.8 ± 3.6	33.7 ± 4	33.7 ± 3.9	0.897
***I Class (%)***	57.7	76.9	73.8	0.026
***II Class (%)***	38.5	15.7	19.4	
***≥III Class (%)***	3.8	7.5	6.9	
***Waist circumference (cm)***	120 ± 10	119 ± 12	119 ± 12	0.624
***Hip circumference (cm)***	118 ± 10	19 ± 10	119 ± 10	0.293
***SBP (mmHg)***	127 ± 15	130 ± 15	129 ± 15	0.817
***DBP (mmHg)***	76 ± 6	77 ± 9	77 ± 9	0.863
***Creatinine (mg/dL)***	1.0 ± 0.2	1.1 ± 0.4	1.1 ± 0.4	0.668
***GOT (IU/L)***	20 ± 7	22 ± 10	21 ± 10	0.487
***GPT (IU/L)***	20 ± 11	21 ± 12	21 ± 12	0.708

ACE-i: angiotensin-converting enzyme inhibitors; ARBs: angiotensin receptor blockers; BMI: body mass index; COPD: chronic obstructive pulmonary disease; DBP: diastolic blood pressure; GOT: serum glutamic oxaloacetic transaminase; GPT: glutamic pyruvic transaminase; HF: heart failure; IQR: inter quartile range; MI: myocardial infarction; PAD: peripheral artery disease; PPI: proton pump inhibitors; SBP: systolic blood pressure; SSRI: selective serotonin reuptake inhibitor; TIA: transient ischemic attack.

**Table 2:** Sensitivity, specificity and predictive values of urinary Dipstick (vs. *trough plasma concentrations*) in patients with atrial fibrillation and obesity.

	Value	95% Confidence Interval
***Sensitivity (%)***	99.24	95.82–99.98
***Specificity (%)***	6.89	0.85–22.76
***Positive Likelihood Ratio***	1.07	0.96–1.18
***Negative Likelihood Ratio***	0.11	0.01–1.18
***Positive Predictive Value (%)***	82.8	81.33–84.18
***Negative Predictive Value (%)***	66.67	15.79–95.52

PLR: positive likelihood ratio; NLR: negative likelihood ratio; PPV: positive predictive value; NPV: negative predictive value; CI: confidence interval.

**Table 3:** Characteristics of patients according to false/true positive status.

	True Positive (n: 130)	False Positive (n: 27)	*p*-Value
***Age (mean)***	73.8 ± 9.0	70.5 ± 9.7	0.405
***Female (%)***	33.6	40.0	0.537
***Hypertension (%)***	93.9	100.0	0.205
***Diabetes (%)***	38.2	44.0	0.584
***Previous MI (%)***	22.1	20.0	0.812
***Previous stroke/TIA (%)***	14.5	32.0	0.034
***Smoke (%)***	12.2	8.0	0.546
***Ex-smoke (%)***	45.0	40.0	0.642
***COPD (%)***	19.1	24.0	0.572
***PAD (%)***	6.1	12.0	0.292
***HF (%)***	26.0	12.0	0.133
***Liver disease (%)***	11.5	12.0	0.937
***Cancer (%)***	17.6	12.0	0.494
***BMI < 35 (%)***	77.9	60.0	0.059
***BMI ≥ 35 (%)***	22.1	40.0	
***CHA_2_DS_2_-VASc Score***	3.9 ± 1.6	4.1 ± 1.6	0.977
***HAS-BLED score***	1.2 ± 0.7	1.1 ± 0.7	0.591
***Therapy***			
***Antiplatelet (%)***	4.6	0.0	0.275
***ACE inhibitors/ARBs (%)***	73.1	72.0	0.912
***Nitro-derivates (%)***	3.8	8.0	0.355
***Beta-blockers (%)***	72.5	80.0	0.436
***Digoxin (%)***	7.6	16.0	0.317
***Antiarrhythmics (%)***	23.4	16.0	0.413
***Amiodarone (%)***	7.1	0.0	0.179
***Statins (%)***	73.3	80.0	0.481
***Ezetimibe (%)***	22.9	32.0	0.331
***SSRI (%)***	7.8	4.2	0.532
***Diuretics (%)***	62.6	72.0	0.369
***PPI (%)***	62.6	52.0	0.320
***Allopurinol (%)***	17.6	16.0	0.850
***Clinical Features***			
***BMI (continuous)***	33.3 ± 3.8	35.3 ± 4.6	0.317
***Waist circumference (cm)***	118.9 ± 11.8	121.5 ± 11.7	0.588
***Hip circumference (cm)***	118.6 ± 10.0	122.0 ± 12.4	0.327
***SBP (mmHg)***	130.0 ± 15.7	126.2 ± 11.0	0.214
***DBP (mmHg)***	77.4 ± 8.9	76.0 ± 8.7	0.864
***GOT (IU/L)***	21.7 ± 9.7	19.7 ± 8.3	0.625
***GPT (IU/L)***	20.5 ± 12.0	20.3 ± 11.2	0.515
***Trough plasma concentrations (ng/mL)***	88.1 ± 57.8	13.0 ± 14.9	0.588
***Peak plasma concentrations (ng/mL)***	202.1 ± 98.4	117.9 ± 102.5	0.327

ACE-i: angiotensin-converting enzyme inhibitors; ARBs: angiotensin receptor blockers; BMI: body mass index; COPD: chronic obstructive pulmonary disease; DBP: diastolic blood pressure; GOT: serum glutamic oxaloacetic transaminase; GPT: glutamic pyruvic transaminase, HF: heart failure; IQR: inter quartile range; MI: myocardial infarction; PAD: peripheral artery disease; PPI: proton pump inhibitors; SBP: systolic blood pressure; SSRI: selective serotonin reuptake inhibitor; TIA: transient ischemic attack.

**Table 4:** Sensitivity, specificity and predictive value of urinary Dipstick in patients with atrial fibrillation and obesity according to Factor IIa and Factor Xa inhibitors.

	Anti-Xa Inhibitors		Dabigatran	
	Value	95% CI	Value	95% CI
***Sensitivity (%)***	100	79.4–100	99.1	95.3–99.9
***Specificity (%)***	10.0	0.25–44.5	5.3	0.13–26.0
***PLR***	1.1	0.90–1.37	1.05	0.94–1.16
***NLR***	0		0.16	0.01–2.5
***PPV (%)***	64.0	59.1–68.6	86.3	85.0–87.6
***NPV (%)***	100	2.5–100	50	6.1–93.9

PLR: positive likelihood ratio; NLR: negative likelihood ratio; PPV: positive predictive value; NPV: negative predictive value; CI: confidence interval.

**Table 5:** Sensitivity, specificity, predictive values and accuracy of urinary dipstick in patients with AF and obesity according to the trough plasma concentrations cut-off of 30 ng/mL.

	Trough Plasma Concentrations 30 ng/mL	
	Value	95% CI
***Sensitivity (%)***	99.1	95.4–100
***Specificity (%)***	4.7	0.6–16.2
***PLR***	1.04	0.97–1.12
***NLR***	0.18	0.02–1.91
***PPV (%)***	74.5	73.2–75.8
***NPV (%)***	66.7	15.7–95.6
***Accuracy (%)***	74.4	66.9–81

PLR: positive likelihood ratio; NLR: negative likelihood ratio; PPV: positive predictive value; NPV: negative predictive value; CI: confidence interval.

## Data Availability

The data that support the findings of this study are available from the corresponding author upon reasonable request.

## Supplementary Materials

The following supporting information can be downloaded at: https://www.mdpi.com/article/10.3390/jcm15020466/s1, Supplementary Methods: Determination of dabigatran plasma concentration, Determination of rivaroxaban, apixaban and edoxaban plasma concentration; Table S1: Obesity category based on BMI (Body Mass Index); Table S2: The trough and peak plasma concentrations range of direct oral anticoagulants; Table S3: Sensitivity, specificity and predictive values of urinary dipstick in patients with atrial fibrillation and obesity according to peak plasma concentrations; Table S4: Sensitivity, specificity and predictive value of urinary dipstick in patients with atrial fibrillation and obesity according to each DOAC; Table S5: Paired plasma and urine samples are available for analysis according to each direct oral anticoagulant; Table S6: Subgroup analyses of sensitivity, specificity and predictive values of urinary dipstick in patients with atrial fibrillation and obesity on trough plasma concentration threshold ≥30 ng/mL (Panel A) and on peak plasma concentration (Panel B) according to anti-Xa anti-IIa inhibitors; Table S7: Subgroup analyses of sensitivity, specificity and predictive values of urinary Dipstick in patients with atrial fibrillation and obesity according to obesity degree; Table S8: True positive, false positive, true negative and false negative according to obesity class and type of DOAC (Factor Xa or dabigatran); Table S9: Sensitivity, specificity and predictive values of urinary dipstick in patients with atrial fibrillation and obesity according to weight ≥120 kg or <120 kg; Table S10: Sensitivity, specificity and predictive values of urinary dipstick in patients with atrial fibrillation and obesity according to creatinine clearance; Table S11: Sensitivity, specificity and predictive values of urinary dipstick in patients with atrial fibrillation and obesity according to standard or low dose of direct oral anticoagulants.
