# Supplementary material for: Diagnostic Performance of a DOAC Urine Dipstick in Obese Outpatients with Atrial Fibrillation: Comparison with Plasma Concentrations
Source: J Clin Med. 2026 Jan 7;15(2):466. doi: 10.3390/jcm15020466 (PMC12841614; doi:10.3390/jcm15020466)
Supplement: Supplementary file 1 [file jcm-15-00466-s001.zip › jcm-4053166-supplementary.pdf]

Diagnostic performance of a DOAC urine dipstick in obese outpatients with atrial fibrillation: comparison with plasma concentrations

Supplementary file

Supplementary Methods.

Table S1. Obesity category based on BMI (Body Mass Index).

Table S2. Trough and peak plasma concentrations range of direct oral anticoagulants.

Table S3. Sensitivity, specificity and predictive values of urinary dipstick in patients with atrial fibrillation and obesity according to peak plasma concentrations

Table S4. Sensitivity, specificity and predictive value of urinary dipstick in patients with atrial fibrillation and obesity according to each DOAC.

Table S5. Paired plasma and urine samples available for analysis according to each direct oral anticoagulant.

Table S6. Subgroup analyses of sensitivity, specificity and predictive values of urinary dipstick in patients with atrial fibrillation and obesity on trough plasma concentration threshold ≥30ng/ml (Panel A) and on peak plasma concentration (Panel B) according to anti-Xa anti-IIa inhibitors.

Table S7. Subgroup analyses of sensitivity, specificity and predictive values of urinary Dipstick in patients with atrial fibrillation and obesity according to obesity degree.

Table S8. True positive, false positive, true negative and false negative according to obesity class and type of DOAC (Factor Xa or dabigatran).

Table S9. Sensitivity, specificity and predictive values of urinary dipstick in patients with atrial fibrillation and obesity according to weight ≥120kg or <120kg.

Table S10. Sensitivity, specificity and predictive values of urinary dipstick in patients with atrial fibrillation and obesity according to creatinine clearance.

Table S11. Sensitivity, specificity and predictive values of urinary dipstick in patients with atrial fibrillation and obesity according to standard or low dose of direct oral anticoagulants.

Supplementary Methods

Determination of dabigatran plasma concentration

The plasma concentration of dabigatran was measured using the diluted thrombin time (dTT) assay, which is the test of choice for measuring the plasma concentration of dabigatran. To reduce the over-sensitivity of the test to thrombin inhibitors, patient plasma was diluted with a pool of normal plasma. The values were expressed as ng/mL and the results are obtained by interpolating the patient's chromogenic activity on a dose-response curve obtained using calibrator plasmas at known drug concentration. The assay was performed using Siemens reagents, certified calibrators and specific controls for dabigatran on the BCS Xp automated coagulometer (Siemens)(1, 2).

Determination of rivaroxaban, apixaban and edoxaban plasma concentration

Plasma concentrations of anti-FXa drugs (rivaroxaban, apixaban, edoxaban) were measured by anti-FXa chromogenic substrate assay, calibrated for each specific drug. The test reflects the ability of plasma to inhibit FXa. It was performed by mixing the patient's plasma with an excess amount of FXa. A specific chromogenic substrate was then added to measure residual FXa. The lower the residual FXa, the higher the drug concentration. Values were expressed as ng/mL and the results were obtained by interpolating the patient's chromogenic activity on a dose-response curve obtained using calibrators at known drug concentration. The assay was performed on a BCS Xp automated coagulometer (Siemens Healthcare Diagnostics, Marburg, Germany). Using Siemens reagents, certified calibrators and specific controls for rivaroxaban, apixaban and edoxaban on the BCS Xp automated coagulometer with internal quality controls were used, which allowed accurate quantification from 0 ng/mL upward, as per the manufacturer’s specifications. No lower limit of quantification was reached in the preset dataset, and all measured values were used in the statistical analysis without substitution or exclusion (1, 3).

Table S1. Obesity category based on BMI (Body Mass Index).

**Obesity category:**	**BMI (kg/m²)**
• I class	30.0-34.9
• II class	35-39.9
• III class	≥40

Table S2. Trough and peak plasma concentrations range of direct oral anticoagulants.

DOAC	Dose	Trough concentration range (ng/ml)	Peak concentration range (ng/ml)
Dabigatran	150mg BID	61-143	117-275
	110mg BID		
Rivaroxaban	20mg	6-239	22-535
	15mg		
Apixaban	5mg BID	41-230	91-321
	2,5mg BID	34-162	69-221
Edoxaban	60mg	19-62	120-245
	30mg	15-45	60-120

DOAC: direct oral anticoagulant; bid: bis in die

Table S3. Sensitivity, specificity and predictive values of urinary dipstick in patients with atrial fibrillation and obesity according to peak plasma concentrations

	**Value**	**95% Confidence Interval**
Sensitivity (%)	97.8	93.7-99.5
Specificity (%)	0	0-15.4
Positive Likelihood Ratio	0.98	0.95-1.0
Negative Likelihood Ratio	-	-
Positive Predictive Value (%)	85.9	85.6-86.2
Negative Predictive Value (%)	0	-

Table S4. Sensitivity, specificity and predictive value of urinary dipstick in patients with atrial fibrillation and obesity according to each DOAC.

	**Edoxaban**		**Rivaroxaban**		**Apixaban**	
	**Value**	**95% CI**	**Value**	**95% CI**	**Value**	**95% CI**
Sensitivity (%)	100	8.05-100	100	89.1-100	98.5	91.8-99.9
Specificity (%)	0	0-40.9	33.3	0.84-90.6	0	0-33.6
PLR	1	1-1	1.5	0.67-3.3	0.98	0.96-1.01
NLR	-	-	0	-	-	-
PPV (%)	70.8	70.8-70.8	94.1	87.78-97.26	87.8	87.5-88.15
NPV (%)	-	-	100	2.5-100	0	-

PLR: positive likelihood ratio; NLR: negative likelihood ratio; PPV: positive predictive value; NPV: negative predictive value; CI: confidence interval; AUC: area under curve

Table S5. Paired plasma and urine samples available for analysis according to each direct oral anticoagulant.

Drugs		N (%)	N True positive (%)	N False positive (%)	N False negative (%)	N True negative (%)
**Dabigatran**	trough	26 (100.0)	16 (61.5)	9 (34.6)	0 (0.0)	1(3.9)
	peak	26 (100.0)	15 (57.7)	10 (38.5)	1 (3.8)	0 (0.0)
**Rivaroxaban**	trough	35 (100.0)	32 (91.4)	2 (5.7)	0 (0.0)	1 (2.9)
	peak	35 (100.0)	31 (88.6)	3 (8.6)	1 (2.8)	0 (0.0)
**Apixaban**	trough	75 (100.0)	65 (86.7)	9 (12.0)	1 (1.3)	0 (0.0)
	peak	74 (100.0)	67 (90.5)	6 (8.1)	1 (1.4)	0 (0.0)
**Edoxaban**	trough	24 (100.0)	17 (70.8)	7 (29.2)	0 (0.0)	0 (0.0)
	peak	24 (100.0)	18 (75.0)	6 (25.0)	0 (0.0)	0 (0.0)

Table S6. Subgroup analyses of sensitivity, specificity and predictive values of urinary dipstick in patients with atrial fibrillation and obesity on trough plasma concentration threshold ≥30ng/ml (Panel A) and on peak plasma concentration (Panel B) according to anti-Xa anti-IIa inhibitors.

Panel A

	**anti-Xa**		**anti-IIa**	
	**Value**	**95% CI**	**Value**	**95% CI**
Sensitivity (%)	100.00	82.3-100.0	98.9	94.5-99.9
Specificity (%)	14.28	0.36-57.9	2.8	0.1-14.9
PLR	1.16	0.86-1.57	1.0	0.9-1.1
NLR	0.0	-	0.3	0.0-5.5
PPV (%)	76.0	70.0-81.1	74.2	73.1-81.1
NPV (%)	100.0	-	50.0	6.0-93.9

Panel B

	**anti-Xa**		**anti-IIa**	
	**Value**	**95% CI**	**Value**	**95% CI**
Sensitivity (%)	94.1	71.3-99.8	98.3	94.0-99.8
Specificity (%)	0.0	0.0-33.6	0.0	0.0-21.8
PLR	0.94	0.83-1.0	0.98	0.9-1.1
NLR	0.0	-	-	-
PPV (%)	64.0	61.2-66.6	88.8	82.2-93.6
NPV (%)	0.0	-	0.0	-

PLR: positive likelihood ratio; NLR: negative likelihood ratio; PPV: positive predictive value; NPV: negative predictive value; CI: confidence interval

Table S7. Subgroup analyses of sensitivity, specificity and predictive values of urinary Dipstick in patients with atrial fibrillation and obesity according to obesity degree.

**Obesity class**				
	**I**		**≥II**	
	**Value**	**95% CI**	**Value**	**95% CI**
Sensitivity (%)	99.0	94.7-99.9	100	88.0-100
Specificity (%)	0.0	0.0-20.6	15.4	1.9-45.5
PLR	0.99	0.97-1.01	1.18	0.94-1.49
NLR	-	-	0.0	-
PPV (%)	86.2	85.9-86.4	69.0	52.9-82.4
NPV (%)	0.0	-	72.5	67.6-76.9
AUC	0.49	0.40-0.59	0.58	0.41-0.72

PLR: positive likelihood ratio; NLR: negative likelihood ratio; PPV: positive predictive value; NPV: negative predictive value; CI: confidence interval; AUC: area under curve.

Table S8. True positive, false positive, true negative and false negative according to obesity class and type of DOAC (Factor Xa or dabigatran).

Obesity	Factor Xa	N (%)	N True positive (%)	N False positive (%)	N False negative (%)	N True negative (%)
**Class l**	trough	103 (100.0)	91	11	1	0 (0.0)
	peak	103 (100.0)	93	9	1	0 (0.0)
**Class >2**	trough	31 (100.0)	23	7	0 (0.0)	1
	peak	30 (100.0)	23	6	1	0 (0.0)
**Obesity**	**Dabigatran**	**N (%)**	**N True positive (%)**	**N False positive (%)**	**N False negative (%)**	**N True negative (%)**
**Class l**	trough	15 (100.0)	10	5	0 (0.0)	0 (0.0)
	peak	15 (100.0)	11	4	0 (0.0)	0 (0.0)
**Class >2**	trough	11 (100.0)	6	4	0 (0.0)	1
	peak	11 (100.0)	5	5	1	0 (0.0)

Table S9. Sensitivity, specificity and predictive values of urinary dipstick in patients with atrial fibrillation and obesity according to weight ≥120kg or <120kg.

	**Weight ≥120kg**		**Weight <120kg**	
	**Value**	**95% CI**	**Value**	**95% CI**
Sensitivity (%)	100	71.51-100	99.07	94.90-99.98
Specificity (%)	0	0-70.76	5.13	0.63-17.32
PLR	1	1-1	1.04	0.97-1.13
NLR	-	-	0.18	0.02-1.95
PPV (%)	78.57	49.20-95.34	74.13	72.66-75.54
NPV (%)	-	-	66.67	15.72-95.54

PLR: positive likelihood ratio; NLR: negative likelihood ratio; PPV: positive predictive value; NPV: negative predictive value; CI: confidence interval.

Table S10. Sensitivity, specificity and predictive values of urinary dipstick in patients with atrial fibrillation and obesity according to creatinine clearance.

Creatinine clearance with CKD-EPI formula						
	**≤G3**		**G2**		**G1**	
	**Value**	**95% CI**	**Value**	**95% CI**	**Value**	**95%CI**
Sensitivity (%)	98.1	89.7-99.9	100	93.8-100	100	80.5-100
Specificity (%)	0.0	0.0-60.2	5.6	0.14-27.3	20.0	0.51-71.6
PLR	0.98	0.94-1.02	1.06	0.95-1.18	1.25	0.81-1.94
NLR	-	-	0.0	-	0.0	-
PPV (%)	92.7	92.5-93.0	77.33	75.3-79.2	80.95	73.3-86.8
NPV (%)	0.0	-	100	2.5-100	100	2.5-100
Accuracy (%)	91.1	80.4-97	77.6	66.6-86.4	81.8	59.7-94.8

Table S11. Sensitivity, specificity and predictive values of urinary dipstick in patients with atrial fibrillation and obesity according to standard or low dose of direct oral anticoagulants.

	Standard dose		Low dose	
	**Value**	**95% CI**	**Value**	**95% CI**
Sensitivity (%)	99.2	95.4-100	100	75.3-100
Specificity (%)	7.7	0.9-25.1	0.0	0-84.2
PLR	1.07	0.96-1.2	1	1-1
NLR	0.11	0.01-1.16	-	-
PPV (%)	83.1	81.5-84.6	86.7	86.7-86.7
NPV (%)	66.7	15.8-95.5	-	-
Accuracy (%)	82.7	75.6-88.5	86.7	59.5-98.3

Reference

1. Amiral J, Dunois C, Amiral C, Seghatchian J. Anti-Xa bioassays for the laboratory measurement of direct Factor Xa inhibitors in plasma, in selected patients. Transfus Apher Sci. 2016;55(2):249-61.
2. Dunois C. Laboratory Monitoring of Direct Oral Anticoagulants (DOACs). Biomedicines. 2021;9(5).
3. Studt JD, Alberio L, Angelillo-Scherrer A, Asmis LM, Fontana P, Korte W, et al. Accuracy and consistency of anti-Xa activity measurement for determination of rivaroxaban plasma levels. J Thromb Haemost. 2017;15(8):1576-83.
